# Nanofluidic memristor based on the elastic deformation of nanopores with nanoparticle adsorption

**DOI:** 10.1093/nsr/nwad216

**Published:** 2023-08-11

**Authors:** Xi Zhou, Yuanyuan Zong, Yongchang Wang, Miao Sun, Deli Shi, Wei Wang, Guanghua Du, Yanbo Xie

**Affiliations:** Department of Chemistry and Chemical Engineering, Northwestern Polytechnical University, Xi’an 710072, China; School of Physical Science and Technology, Northwestern Polytechnical University, Xi’an 710072, China; School of Physical Science and Technology, Northwestern Polytechnical University, Xi’an 710072, China; School of Aeronautics and Institute of Extreme Mechanics, Northwestern Polytechnical University, Xi’an 710072, China; School of Physical Science and Technology, Northwestern Polytechnical University, Xi’an 710072, China; School of Physical Science and Technology, Northwestern Polytechnical University, Xi’an 710072, China; Institute of Modern Physics, Chinese Academy of Sciences, Lanzhou 730000, China; School of Physical Science and Technology, Northwestern Polytechnical University, Xi’an 710072, China; School of Aeronautics and Institute of Extreme Mechanics, Northwestern Polytechnical University, Xi’an 710072, China

**Keywords:** nanofluidics, memristor, neurocomputing, elastic deformation

## Abstract

The memristor is the building block of neuromorphic computing. We report a new type of nanofluidic memristor based on the principle of elastic strain on polymer nanopores. With nanoparticles absorbed at the wall of a single conical polymer nanopore, we find a pinched hysteresis of the current within a scanning frequency range of 0.01–0.1 Hz, switching to a diode below 0.01 Hz and a resistor above 0.1 Hz. We attribute the current hysteresis to the elastic strain at the tip side of the nanopore, caused by electrical force on the particles adsorbed at the inner wall surface. Our simulation and analytical equations match well with experimental results, with a phase diagram for predicting the system transitions. We demonstrate the plasticity of our nanofluidic memristor to be similar to a biological synapse. Our findings pave a new way for ionic neuromorphic computing using nanofluidic memristors.

## Teaser

Adsorption of nanoparticles in a polymer nanopore causes frequency-dependent memory effects by voltage induced elastic strain. The successful emulating of adaptive learning opens a new approach for neuromorphic computing.

## INTRODUCTION

As the fourth fundamental electronic element [1,2], the memristor was thought to be crucial for the next generation of artificial intelligence [3–5]. A non-volatile memristor processes and stores information by resistance switches, resulting in fast and efficient neuromorphic computing, similar to biological synapses. Since Hewlett-Packard found the semiconductor by nanojunction of Ti/TiO_*x*_ [6], many of the new thin-film materials, particularly two-dimensional materials, have been discovered to have memory effects [7]. The signal transmission of most existing memristors is based on electrons using inorganic or organic semiconductors [8–10]. However, the emergence of nanofluidic memristors opens a new window for mimicking a biological synapse, as the signal transmission relies on the ionic transport in a water system. Recently, it was reported that memristors can be inserted into different microfluidic or nanofluidic devices, which could significantly help in mimicking an artificial synapse in the future [11–13].

There are only a few types of ionic nanofluidic memristors, and most of the mechanisms rely on ion-ion or ion-surface interactions, such as surface adsorption and desorption [14], ion concentration polarization [15] and electro-activated transitions of liquids in the nanochannel [16,17], that have recently been proposed as micro/nanofluidic memristors. Recently, the dissociation of Bjerrum ion pairs under an electrical field has been proposed for constructing electrolyte ionic memristors [11], and proved by experiments [13]. A chemical nanofluidic memristor recently emerged that works for both computing and biomedical applications [18]. However, the development of water-based memristors still lags far behind that of solid-state film memristors. Developments in nanofluidic memristors may significantly aid in mimicking the brain by hydrated ion transportation.

In this work, we fabricate a nanofluidic memristor based on the principle of elastic strain of a single polymer nanopore that has never been reported before [19], with adsorption of SiO_2_ nanoparticles (NPs) at the tip side of polymer nanopores in the electrolyte solution. We find a clear current hysteresis in a wide range of scanning voltages and frequencies, which gradually becomes an Ohmic resistor as scanning frequencies increase and a diode as frequencies decrease. Contrary to previous work with NP blockade of a digit nanopore at a rigid substrate [20], the conductance with NP additives is even higher than that of the bared nanopore, suggesting that the hysteresis was not caused by the resistance blockade of NPs. We suspect that the resistance switches were caused by the elastic strain of the polymer nanopore, which was activated by the electrical forces of the negatively charged surface of SiO_2_. We investigate the on : off ratio of the system by scanning the frequency and voltage amplitude, presenting the threshold conditions of memristors in a phase diagram. Our numerical simulation and elastic theory both agree well with the experimental results of conductance switches, which may help further optimization of our elastic nanofluidic memristor. Finally, we demonstrate the synaptic plasticity of our nanofluidic memristors, which enables a Hebbian learning process once the time intervals between stimulus spikes are smaller than a threshold value. We believe that our work may pave a way for a new method of building nanofluidic memristors that can be used for neuromorphic computing in water systems.

## RESULTS AND DISCUSSION

We first fabricated a single conical nanochannel with ion track etching technology [21–24]; for more details of the fabrication process and measurements, see the section entitled ‘Methods and Materials’ and Figs S1 and S2 within the online supplementary material. We dispersed SiO_2_ nanoparticles in various concentrations of KCl solution at pH 8.5, and filled the chamber at the base side of the nanochannel. We filled the same KCl solutions without NPs at the tip side. The sedimentation of NPs was observed in 1-M KCl solutions; however, we found that the results only mattered with the absorbed NPs at the tip of the nanochannel. We first electrophoretically drove the NPs in the nanochannel by DC voltage, and operated the cyclic voltammetry (CV) in pure KCl solutions (without NPs in solution; Fig. S3 within the online supplementary material). Without NPs dissolved in solution, we still found a clear current hysteresis after adsorption of NPs. Hence, our devices work well with long-term storage (Fig. S4 within the online supplementary material). We work with polystyrene NPs and CrSe quantum dots, the results of which showed similar current hystereses (Fig. S5 within the online supplementary material), possibly due to the comparable surface properties of NPs, as discussed below.

**Figure 1.**
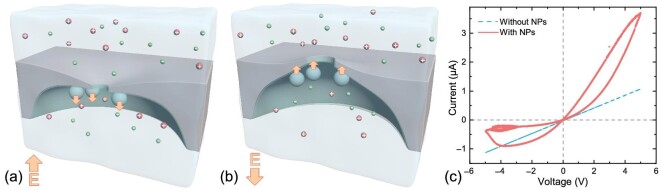
Schematic pictures of the elastic strain at the tip of a single conical nanochannel, causing conductance switches under the negative (a) and positive (b) electrical fields with an additive of SiO_2_ nanoparticles. (c) We found a clear current hysteresis after the addition of NPs (red solid line); however, the current responses obey Ohmic’s law in pure KCl solutions before the addition of NPs.

A typical I-V curve with the addition of NPs in 1-M KCl is shown in Fig. 1(c). We applied a triangle wave of the voltage scanning with a frequency of 0.05 Hz and an amplitude voltage of 5 V. The dashed line in Fig. 1(c) shows the Ohmic current responses and current amplitude in pure electrolyte solutions from 1 V scanning before the addition of NPs, while a typical I-V curve (red solid line) after the addition of NPs shows a clear pinched current hysteresis. Surprisingly, the conductance is overall larger than that in pure 1-M KCl solutions before adding NPs (dashed line in Fig. 1(c)), besides the conductance increases as applied voltage instead of a constant. Such an increase in conductance is against our intuition since the electrophoresis of NPs toward the tip side of the nanopore may induce a resistance increase. Besides, the amplitude of the current is asymmetric in positive and negative voltages, which is never observed in 1-M KCl where bulk conductance is dominant. We investigated the effects of salt concentration and observed a pinched current hysteresis in electrolyte solutions above 0.1 M (Fig. S6 within the online supplementary material). The hysteresis vanished below 10-mM KCl solutions since the conduction was dominated by surface conduction, so changes in the pore tip geometry would not affect system conduction.

Similar current hystereses were observed in different salt concentrations and sizes of NPs (Figs S6 and S10 within the online supplementary material); however, they vanished in concentrations lower than 1-mM KCl. In addition, we found that the current hysteresis disappeared with work in pH = 3.5 at the same condition, which is close to the isoelectric point of SiO_2_ NPs (Fig. S9 within the online supplementary material). Thus, we suspect that it was caused by the electrical force of negatively charged NPs, inducing strain at the tip of the conical nanopore and thus increasing the conductance. In particular, the etching-stopping fabrication of NPs may leave a thin layer of polymer film at the tip for the adsorption of NPs, as shown in Fig. 1(a) and (b) [21]. Unfortunately, it is difficult to characterize the deformation from the tip side, due to the random distribution of pores and the small size of the blister.

**Figure 2.**
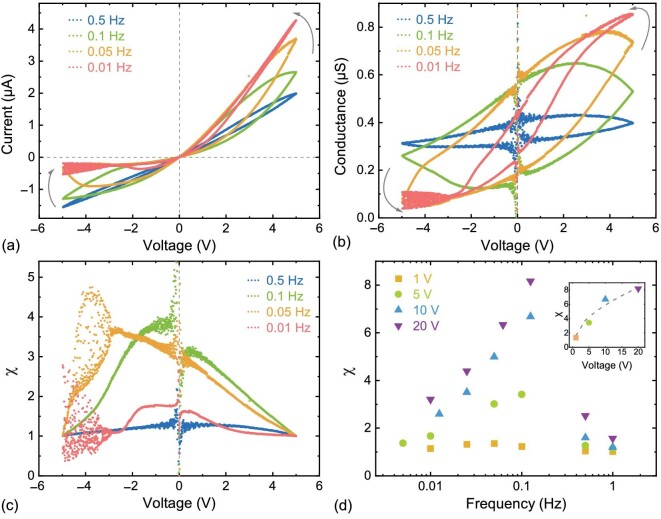
(a) I–V curves of the polymer nanopores with the addition of NPs at different scanning frequencies. (b) The conductance switches as a function of the applied bias voltage for various scanning frequencies. (c) The on : off ratio, calculated by the conductance ratio in forward and reversed scanning directions, nearly always represented a peak value at *V* = 0. (d) The on : off ratio χ|_*V* = 0_ represents peak values close to ∼0.1 Hz, which transits to an Ohmic resistor with increasing *f* but a diode with decreasing *f*, both of which result in a reduction in the on : off ratio.

We first focused on the impact of scanning frequencies *f*. For comparison, we fixed the amplitude of the voltage as a constant at 5 V while increasing *f* from 0.005 to 1 Hz. Our results showed that the current linearly (blue dots in Fig. 2(a)) increases as the bias voltage increases when *f* > 0.5 Hz. As *f* decreases from 0.1 to 0.05 Hz, we found clear current hysteresis loops pinched at 0 V in both polarities of the bias voltage. The current loop is anti-clockwise at a positive bias voltage caused by increasing conductance. At the negative bias voltage, the current hysteresis shows a clockwise loop with a minimum value. When *f* further decreases below 0.01 Hz, the current hysteresis rapidly shrinks. Besides, the system is represented as a diode with a low resistance state at the positive bias voltage and a high resistance state at the negative bias voltage. The ‘diode’-like behavior of current-voltage responses were probably not caused by the surface conduction within electrical double layers, since the surface conduction is negligible in a 1-M KCl electrolyte solution. We attributed this diode-like conductance of the strain at the tip of polymer nanopores to the adsorption of NPs. The current hysteresis for each *f* can be excellently repeated for over 30 cycles (up to 60 cycles in Fig. S7 within the online supplementary material). Besides, we found vibrating signals when the conductance gets close to the minimum value at a negative bias voltage, possibly induced by the motion of NPs within nanopores.

We calculated conductance by *G* = *I*/*V* for a wide range of *f* to present the state transitions. The results showed that the conductance remains nearly a constant, $\sim\! 0.4\, \mu $S at *f* > 0.5 Hz, thus nearly representing an Ohmic system. As *f* transits from 0.1 to 0.02 Hz, the conductance shows clear hysteresis loops and the resistance of the system is correlated with the previous states, representing memory effects. Once *f* < 0.01 Hz, the conductance loops gradually shrink, while the resistance still shows a significant transition, similar to a diode.

To quantitatively evaluate the amplitude of the current hysteresis, we calculate the on : off ratio χ using the conductance in both scanning directions, $\chi =G_{0_-}/G_{0_+}$, where subscripts +, − indicate the polarity of *dG*/*dV* at a certain voltage. Our results in Fig. 2(c) show that the χ|_*V* = 0_ reached a maximum value for nearly all frequencies; thus, we take χ|_*V* = 0_ to evaluate the memory effects of our devices. To avoid singularity points of *G* at *V* = 0, we linearly fit the current within ±0.5 V to obtain $G_{0_-}, G_{0_+}$ to estimate the conductance switches in various frequencies. Our results in Fig. 2(d) indicate that χ reached a maximum value at ∼0.1 Hz.

Besides, we still find a weak current hysteresis (maximum χ of 1.3) at a voltage amplitude of 1 V (Fig. 2(d)) due to the neglected strain of the pore tip. We statistically derived the maximum χ as a function of the voltage amplitude (shown as dots in Fig. 2(d)); current recordings are given in Fig. S8 within the online supplementary material. The solid line indicates the power law of *V*^2/3^ obtained from the elastic strain theory of the pore formation, which will be analytically derived in later elastic theories, in particular when the stress is below a critical threshold value [25]. Our results on the current hysteresis perfectly matched with the definition of the generic memristor defined by Chua in the 1970s [1,2]. We believe that the hysteresis was not caused by electrolysis, as the memory effects had not been observed before adsorption of NPs in any previous work. A few approaches exist that can reduce the voltage amplitude, avoiding electrolysis, like thinning the tip side of the nanochannel, enlarging the surface area of the tip side and charge density on the particles, according to the theoretical analysis presented in the next section.

This is possibly due to the fact that resistance switches were caused by the strain of the tip of the nanopore, but the surface conductance gradually becomes dominant in diluted solutions, resulting in a smaller on : off ratio. In addition, we suspect that the NPs were attached to the inner layer of the polymer nanopore, which possibly has a limited impact on the conductance. We found in experiments that the adsorbed NPs are difficult to remove from the surface. Besides, the deformation of the polymer tip seems closely relevant to the pore tip geometry of the nanochannel, which may induce different types of current hysteresis, as we found in different nanopores. We suspect that we occasionally fabricated a single conical nanopore with a thin flexible layer at the tip side due to the electroosmosis of acid solutions [21]. Thus, different shapes and properties at the tip side may induce different current hystereses, which is possibly a constraint for our current devices. However, it inspired us to explore a new principle for building nanofluidic memristors by electro-activated geometry deformation, once the thin film can be well modulated.

### Theory and simulation

We rationalized the resistance switches by the linear elastic strain theory framework, caused by the electrostatic force on NPs adsorbed at the tip side of the conical nanochannel. The elastic theory successfully predicted the conductance change under external pressure on the membrane [26]. We estimate the electrostatic force acted on the tip of conical polymer nanopores by NPs, expressed as *F* = *N*Σ_*e*_*A_np_E*, where *N* is the number of adsorbed particles calculated by *N* ∼ ρ*A_p_* with the density of NPs ρ and the area of the nanopore tip *A_p_*. Here Σ_*e*_, *A_np_* denoted the surface charge density and surface area of a single nanoparticle. Thus we could derive the pressure applied on the film *p* = *F*/*A_p_*, caused by NPs under an electrical field as follows [27]. The SiO_2_ NPs were negatively charged in pH = 8.5 KCl solution that may lift up the tip of the polymer nanopore under positive bias voltage, finally increasing the conductance of the system, which is consistent with our experimental results. We have


(1) \begin{equation*}
p = \rho _p \Sigma _e A_{np} E.
\end{equation*}

Here we consider a thin layer of polymer pore that is deformable under stress at the tip side of the nanochannel. The strain of a thin layer film can be expressed as *d* = [1 + ε(σ)]*d*_0_, where *d, d*_0_ are the diameters under stress and initial conditions. The strain ε in the equilibrium state can be calculated as ε = (1 − γ^2^)σ/*E_S_*, where γ and *E_S_* are Poisson’s ratio and Young’s modulus of the thin layer at the surface of the polymer film. The stress σ_*e*_ at the equilibrium state can be calculated using the equation


(2) \begin{equation*}
\sigma _e (n)^3-\sigma _0 \sigma _e (n)^2 - \frac{E_S p(n)^2 A_p}{6h^2 (1-\gamma )^2}= 0,
\end{equation*}

where subscript *n* indicates the stress at the scanning step of *n* and σ_0_ is the residual stress of the polymer film that we neglected in our calculation. For a conical nanopore, we know the conductance *G* = κ*Dd*/4*L*, where *D* and *d* are the diameters of the base and tip side of the conical nanopore, respectively. Thus, we could derive the change of conductance as *G*/*G*_0_ = *d*/*d*_0_, since the base side remained a constant. By stepwise increasing the applied voltage on the tip of the conical polymer nanochannel, we consider the step load Δ*p* to be a constant. According to the time-dependent load strain behavior, we can derive the time-dependent strain by an exponential rising as follows, neglecting a creeping process [28,29]:


(3) \begin{eqnarray*}
\Delta \varepsilon _t (n)&=&\bigg (\frac{1-\gamma ^2}{E_S}\bigg )[\sigma _e (n)-\sigma _t (n-1)]\nonumber\\
&&\quad \quad -(1-e^{-\Delta t/\tau }).
\end{eqnarray*}

Here τ is the relaxation time of strain on the tip of the polymer nanochannel, which we derived from the fitting of conductance switches (∼7.8 s). By equation (3), we found that the strain at the equilibrium state (equation (2)) reached a saturated value at low frequencies (Δ*t* ≫ τ). Thus, the current nearly monotonically increases as the applied voltage either lifts up at positive voltage or depresses the tip side of the nanopore at negative voltage, representing a negligible hysteresis. The negative-voltage-induced depression of the nanopore tip thus has a high resistance state. Finally, we have a diode-like system at low scanning frequencies *f*. When we work in high frequency Δ*t* ≪ τ, Δσ_*t*_(*n*) approaches 0, which indicates that the strain and stress remained a constant and thus in an Ohmic state.

**Figure 3.**
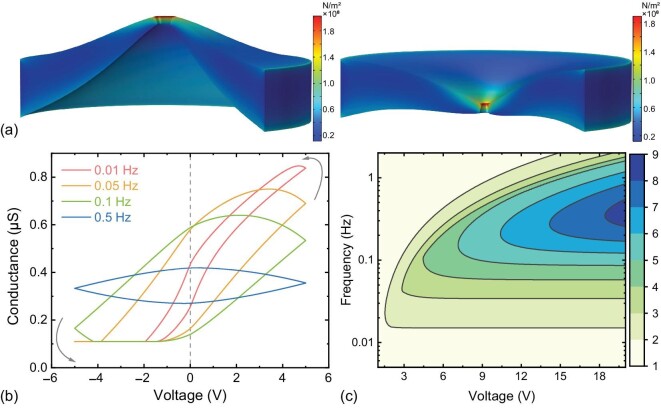
(a) The elastic strain of the pore tip under positive (left) and negative (right) electrical forces. The color bars indicate the stress distribution on the pore tip. (b) Conductance transition with a full scanning cycle of voltage, which matched well with our experimental results. (c) Diagram of the on : off ratio χ|_*V* = 0_ as functions of the voltage amplitude and scanning frequency.

With the time-dependent strain ε_*t*_(*n*) of each scanning step, we derived the elastic strain under a scanning voltage by taking the strain-stress relation in equation (2) as follows. Finally, we could calculate the time-dependent conductance by the strain of the nanopores shown as a solid line in Fig. 3(b), which matched well with our experimental results. We have


(4) \begin{equation*}
\frac{G}{G_0} = 1+ \sum _n \Delta \varepsilon _t (n).
\end{equation*}

We set the Poisson ratio γ = 0.3, Young’s modulus at the surface thin layer of the polymer film *E_s_* = 0.47 GPa, and the mass density to be 1.42 kg/m^3^, where the modulus at the thin surface was reported smaller than the bulk materials [30–32], thus enabling a significant strain of the tip. The initial diameter of the tip side is 8 nm, with the deformable thin layer at the tip side of thickness 10 nm and radius 50 nm. The net charge density of SiO_2_ NPs was 10 mC/m^2^. Under such conditions, only 10 NPs may provide enough elastic stress for a strain at the tip side of the nanochannel. Thus, we derived the conductance transition from theories shown in Fig. 3(b). It needs to be noted that the strain-stress cycles reached stability after 2–3 iterations, since a full cycle of stress often introduced a small amount of strain at the end of a full scanning cycle, depending on the frequencies and amplitude of the voltage. Finally, we calculated a phase diagram for χ as functions of the scanning frequencies *f* and amplitude of the voltage from the elastic theories. With such a phase diagram, we could find the transition of a memristor using nanopores in our experiments. However, the on : off ratio may still be relevant to the absorption of particles, the solution environment and mechanical properties of the thin film at the polymer surface, which we do not focus on here.

To visualize the strain of the thin layer at the tip side of the polymer nanopore, we performed numerical simulations using a linear elastic model; see Fig. 3(a). We set a stress of 5.6 Mpa/V at the inner surface of a conical pore, to match the electrostatic forces caused by the NPs. We found that the tip of the polymer had been pulled up under positive stress, thus inducing the conductance increases. However, the surface sinks at the negative voltage, which shows a minimum value due to the resistance between the pore edges. We derived the time-dependent strain of the pore tip in the simulation, which matched well with the tip size calculated from the experiments for low *f*. More details of the simulation can be found in Fig. S11 within the online supplementary material.

### Plasticity as an artificial synapse

**Figure 4.**
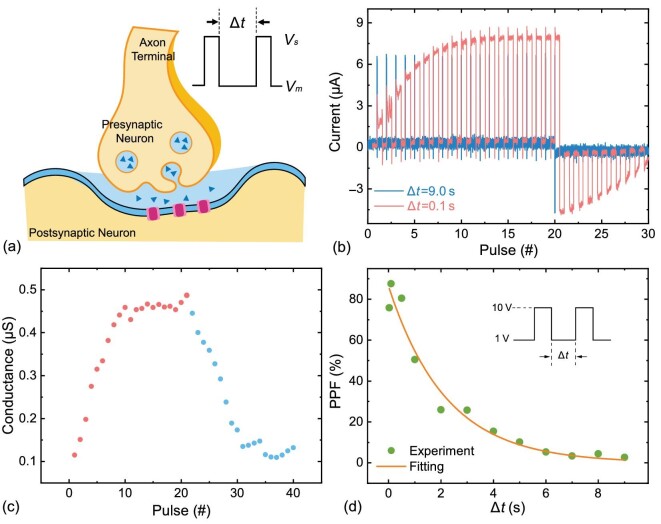
(a) Schematic picture of a biological synapse with spikes in our experiments. (b) The measured current under periodic spikes for the SRDP process in adaptive Hebbian learning. (c) The conductance switches under positive and negative spikes. (d) Paired-pulse facilitation decreases as the time interval between voltage spikes increases, and matches well with an exponential fitting.

As the essential laws of biological neurons, emulating the plasticity of learning is critical. To illustrate the spike-rate-dependent plasticity (SRDP) of our memristor, we applied periodic pulses in a wide range of amplitudes and timing intervals Δ*t* for synaptic adaptive Hebbian learning. We operated periodic stimulating voltages *V_s_* and *V_m_* to measure the conductance state after each stimulating spike. To make a comparison between the potentiation in different spiking intervals, we fixed the amplitude and period of the stimulating voltage as a constant 10 V, while increasing Δ*t* from 0.03 to 9.0 s. Two typical experimental results of measured current with Δ*t* = 0.1 s and Δ*t* = 9.0 s (solid lines) are shown in Fig. 4(b), as a function of the pulse number *N*. With Δ*t* = 0.1 s, shown as the red line in Fig. 4(b), we found that the current rapidly increases and gets saturated at $7.96\,\mu$A after seven cycles, which is about a 4 times larger conductance than the initial state measured by both *V_m_* and *V_s_*. On the contrary, as Δ*t* increases to 9 s, the conductance remains nearly constant (see the blue line in Fig. 4(b)), illustrating the same spike-rate dependence on the synaptic weight as a biological synapse. More results can be found in the online supplementary material.

After consecutive potentiation pulses, we operated depressing spikes to measure the variation of conductance as the process of forgetting information. The results shows that the inhibitory postsynaptic current (blue dots in Fig. 4(c)) exponentially decreases to the level of the initial state. This process can be well repeated under periodic consecutive potentiating and depressing pulses, which emulates long-term potentiation and long-term depression, representing the long-term plasticity characteristics of the synapse. In Fig. 4(d) we summarize the conductance change under the operation of the paired pulse facilitation as a function of the spike interval Δ*t* with potentiation pulses; the results reveal an exponential decrease in the synaptic weight. Frequent and larger stimulus voltages induce a more apparent and faster change in the synaptic weight. All of the above characteristics are close to the SRDP rules of a biological synapse, with however a new principle of the elastic strain of a polymer nanopore.

## CONCLUSIONS

In this work, we report a new type of nanofluidic memristor based on the principle of elastic strain of a polymer nanopore, with adsorption of SiO_2_ nanoparticles on the nanopore inner surface. The nanoparticles bear forces under an external electrical field, deforming the elastic strain at the tip of the nanopores, thus resulting in resistance switches. We found that our memristor transits from a resistor to a memristor and a diode with decreasing frequency, similar to the potassium ion channel in the biological synapse. The feature of a pinched current hysteresis in our experimental results matched well with the fingerprints of the memristor defined by Chua. With potentiation and depressing pulses, we found that it demonstrated a capability of synaptic learning according to Hebbian synaptic adaptation rules. Using linearly elastic strain theory and numerical simulations, we calculated the dynamics of the strain as a function of the electrical field, which matched well with our experimental results. Our work may open new paradigms for building nanofluidic memristors by using an electro-activated elastic strain of soft nanochannels, useful for designing new types of neuromorphic computing devices.

## METHODS AND MATERIALS

### Fabrication of nanopore

The membrane was first irradiated by an energetic heavy Kr ion (∼25 Mev/u) in Langzhou HIRFL, forming a single latent track on each of the 12-${\mu}$m-thick PET foils. Then we mounted the foil between two chambers for chemical etching from one side, while stopping by acid solutions (1 M CHOOH) to protect the other side following the classical fabrication procedure [33,34]. The tip size was controlled in the range of several nanometers, to avoid transporting of the nanoparticles (diameter of 15 nm) through the tip side of the nanopore. More details of the characterizations and devices are given in Fig. S1 within the online supplementary material.

### Electrical measurements

Two Ag/AgCl electrodes were placed in the chambers and connected to a pico-ammeter (Keithley 6482) to record the current under the triangular voltage functional wave. We define the positive bias voltage (electrical field) with an anode at the tip side, while grounded at the base side of the nanopore, for later discussions.

### Finite element modeling

The deformation of the tip side of the nanopore was numerically simulated using a linear elastic model, using equation (2). All simulations were performed when the mesh refinement was sufficient to calculate the stress and strain of the polymer tip. We tested our simulation in the equilibrium state of the elastic strain under a certain applied voltage, which matched well with our experimental results. More details can be found in the online supplementary material.

## Supplementary Material

nwad216_Supplemental_File
