# Supplementary material for: Nanofluidic memristor based on the elastic deformation of nanopores with nanoparticle adsorption
Source: Natl Sci Rev. 2023 Aug 11;11(4):nwad216. doi: 10.1093/nsr/nwad216 (PMC10939365; doi:10.1093/nsr/nwad216)
Supplement: nwad216_Supplemental_File [file nwad216_supplemental_file.pdf]

Nanofluidic memristor by elastic deformation of nanopores with nanoparticles adsorption

Xi Zhou[1*], Yuanyuan Zong[2], Yongchang Wang[2], Miao Sun[3], Deli Shi[2], Wei Wang[2], Guanghua Du[4] and Yanbo Xie[2,3*]

[1]Department of Chemistry and Chemical Engineering

[2]School of Physical Science and Technology

[3]School of Aeronautics and Institute of Extreme Mechanics

Northwestern Polytechnical University, Xi'an, 710072, China

[4]Institute of Modern Physics, Chinese Academy of Sciences, Lanzhou 730000, China

[*]E-mail: zhouxi@nwpu.edu.cn, ybxie@nwpu.edu.cn

June 13, 2023

Contents

1 Methods and Materials **3**

1.1 Fabrication of a single conical track etched nanochannel 3

1.2 I-V measurement with addition of NPs in solution 4

1.3 Measurement with NPs adsorbed nanochannels in pure KCl solution 5

2 Experimental results **6**

2.1 Measurements after long-term storage 6

2.2 Measurements with different types of NPs 7

2.3 Effects of salt solutions 8

2.4 Repeatability in cyclic scanning 9

2.5 Electrochemical measurement in different amplitude of voltage 10

2.6 The impact of pH 11

2.7 The impact of particle size 12

3 Numerical Simulations **13**

1 Methods and Materials

1.1 Fabrication of a single conical track etched nanochannel

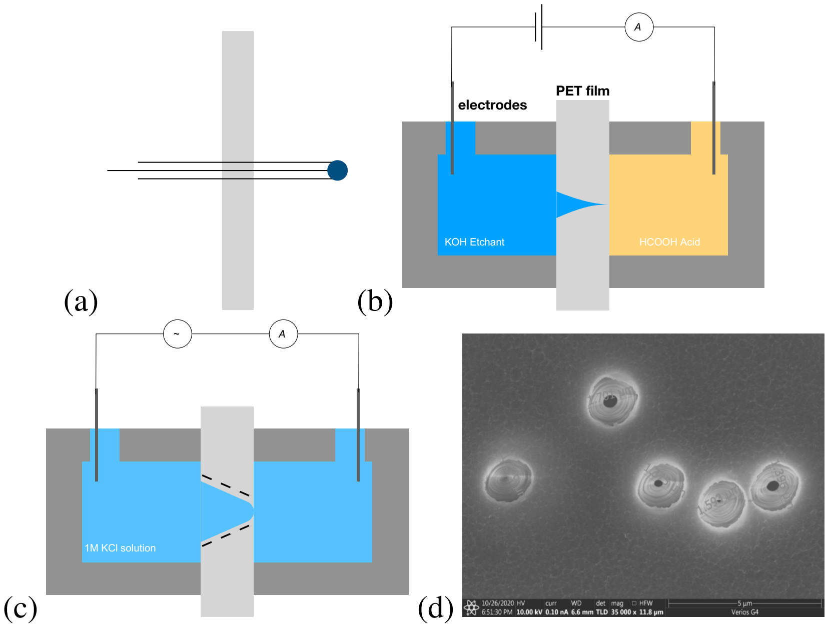

Figure S1: (a) the schematic picture of a single energetic heavy ion bombard the polymer foil, forming a single 1D latent track on the polymer foil. The chemical etching rate of latent track along the trajectory of heavy ion irradiation is over two orders magnitude of the bulk materials, which enables to fabricate 1D nanotube in the PET foil. (b) shows the etching-stopping etching procedure of a conical nanochannel[1, 2]. The 9M KOH solution etched the single single side of the latent track, forming a base side of the nanochannel. We applied a DC bias voltage on the conical nanochannel, and stop the etching process by replacing the KOH stead of HCOOH acid, once we observed an current in the pico-ammeter. The etching process was immersed in a water bath with a constant temperature of 50 degrees. The tip side typically can be controlled within a few nanometers. (c) shows the resistance characterization by 1M KCl electrolyte solution, after the whole etching process. The diameter of base side can be estimated by the etching rate of the PET foil, and calculated the diameter of tip side by resistance of the conical pore as $G = \kappa\pi dD/L$ where d, D, L, κ are the diameter at tip side, base side, length of channel and conductivity of solution respectively. (d) The SEM image of the base side of nanochannel, which were etched after 70 minutes in the water bath.

1.2 I-V measurement with addition of NPs in solution

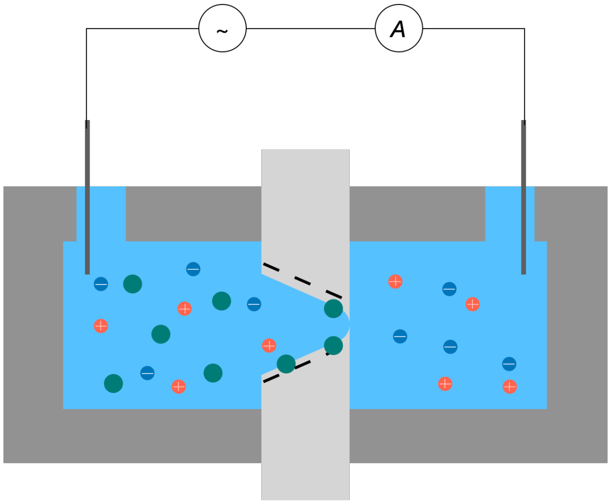

Figure S2: The nanofluidic memristor with addition of SiO_2 nanoparticles (green dots) in the base side, in 1M KCl solution at pH=8.5. Two Ag/AgCl electrodes were placed in two reservoirs, connected with pico-ammeter or electrochemical station for the scanning of voltage and current measurements. The sedimentary of NPs will not affected the performance of devices, once NPs adsorbed at the inner surface of nanochannel. The experimental measurements in pure KCl after NPs adsorbed channel proved our hypothesis as shown in Fig.S3.

1.3 Measurement with NPs adsorbed nanochannels in pure KCl solution

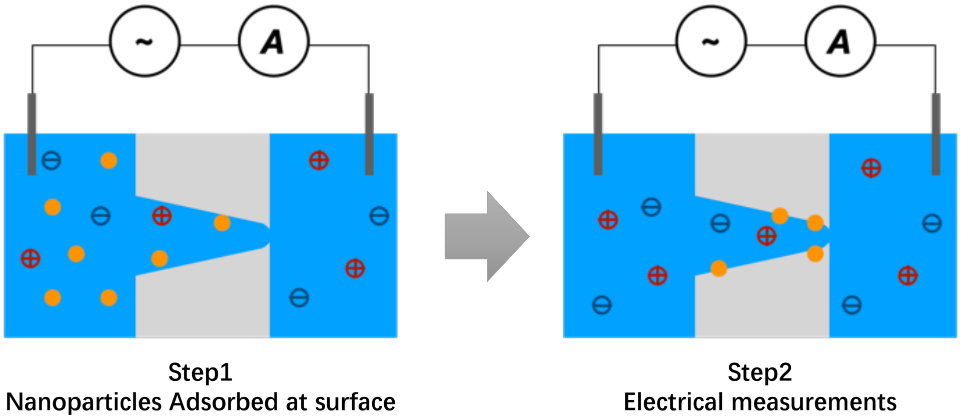

Figure S3: To avoid the effects of sedimentary of NPs in solution, we first work with KCl solutions to make nanoparticles adsorbed at surface by directional electrophoretic (left figure). The solution can be less than 1M KCl, to ensure the adsorption of NPs. Then, we refreshed with the pure KCl solutions (without NPs) and operated the electrical measurements. The results shown in this response letter were operated in the pure KCl solutions, which well repeated the memrisistive behavior in manuscript. The memrisistive characteristics in pure KCl solution indicates the results of memristor can be well repeated in pure KCl solution, avoiding the impacts of NPs sedimentation. The blue and red dots are ions in solution, and yellow dots are nanoparticles.

2 Experimental results

2.1 Measurements after long-term storage

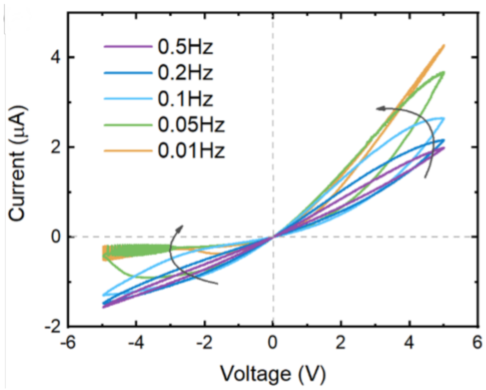

(a)

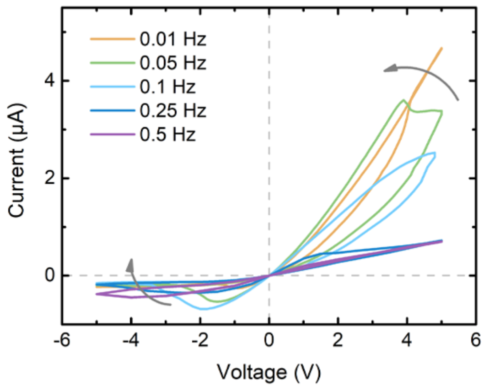

(b)

Figure S4: The I-V curves measured at 2022.December (a) and measurements recently at 2023.April (b). Our nanofluidic devices can repeated well after four months storage. The results in (b) followed the two-step measurement procedures in Fig.S3 in pure 1M KCl solutions, where no SiO_2 nanoparticles dissolved in the 1M KCl aqueous solution. We could still excellent repeated the current hysteresis in long-term storage.

2.2 Measurements with different types of NPs

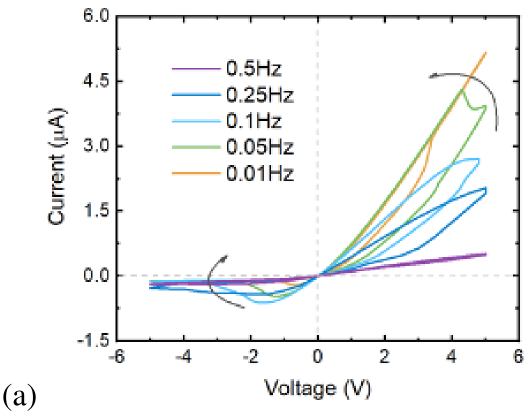

(a)

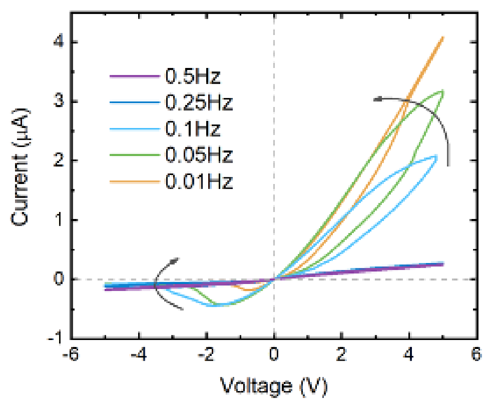

(b)

Figure S5: (a). Measured I-V curve after adsorption of PS NPs. We first operated with DC voltage to drive the PS nanoparticles in the channel. Then we measured with a pure KCl solution (1M) at pH over 8.5 in various range of voltage amplitude. We still found a clear current hysteresis in the system, as similar to the results of SiO_2 NPs. (b). We repeated the electrical measurement process by dissolving CrSe quantum dots instead (pH over 8.5), where we found similar results in the devices. Since the conductance transition was caused by the stress on the tip side of nanochannel, the geometry deformation thus conductance transitions was dominated by the surface charge properties of dielectric NPs. Thus, it matters with the weak surface charge density on NPs, instead of material properties of NPs. The SiO_2, CrSe and PS NPs showed similar current hysteresis in 1M KCl solution.

2.3 Effects of salt solutions

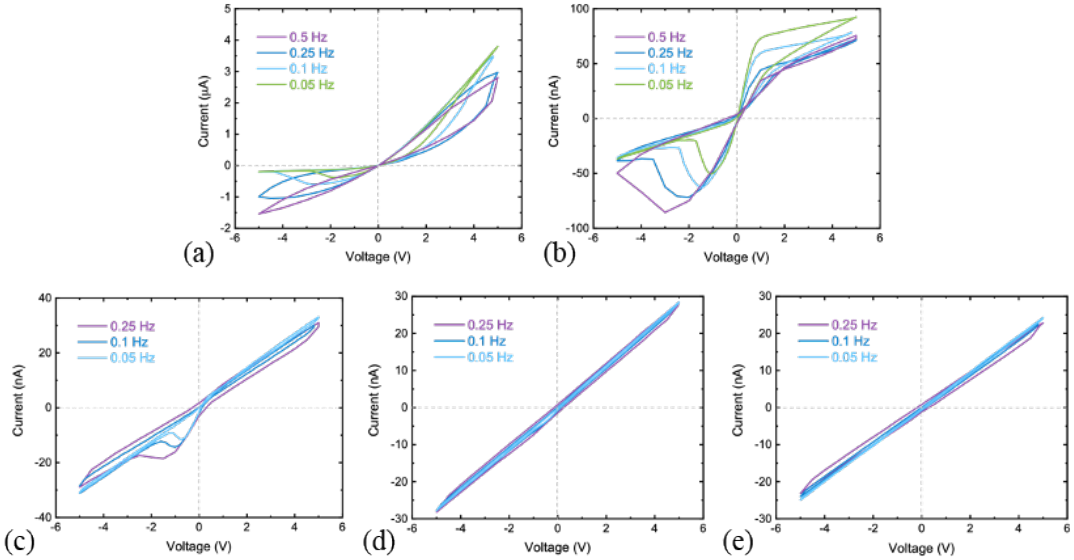

Figure S6: The typical I-V curves followed the two-step measurement procedure, using SiO_2 NPs with various KCl electrolyte solutions at pH=8.5. (a). The results in 1M KCl solutions showed clear current hysteresis which well repeated the previous results again in manuscript. (b). The shape of current hysteresis is even enlarged in 0.1 M KCl solutions, possibly due to the increase of surface charge density of NPs and contribution of surface conduction. However, as (c), (d) and (e) show the ohmic relationship between current and voltage in 0.01 M, 0.001 M, 0.0001 M KCl solution respectively, the current hysteresis gradually vanished as decreases of concentration , which indicates the surface conduction dominated so that the geometry change of pore tips will not affect the system conduction (comparable to the conduction plateau when double layer overlaps, as Ref.[3].

2.4 Repeatability in cyclic scanning

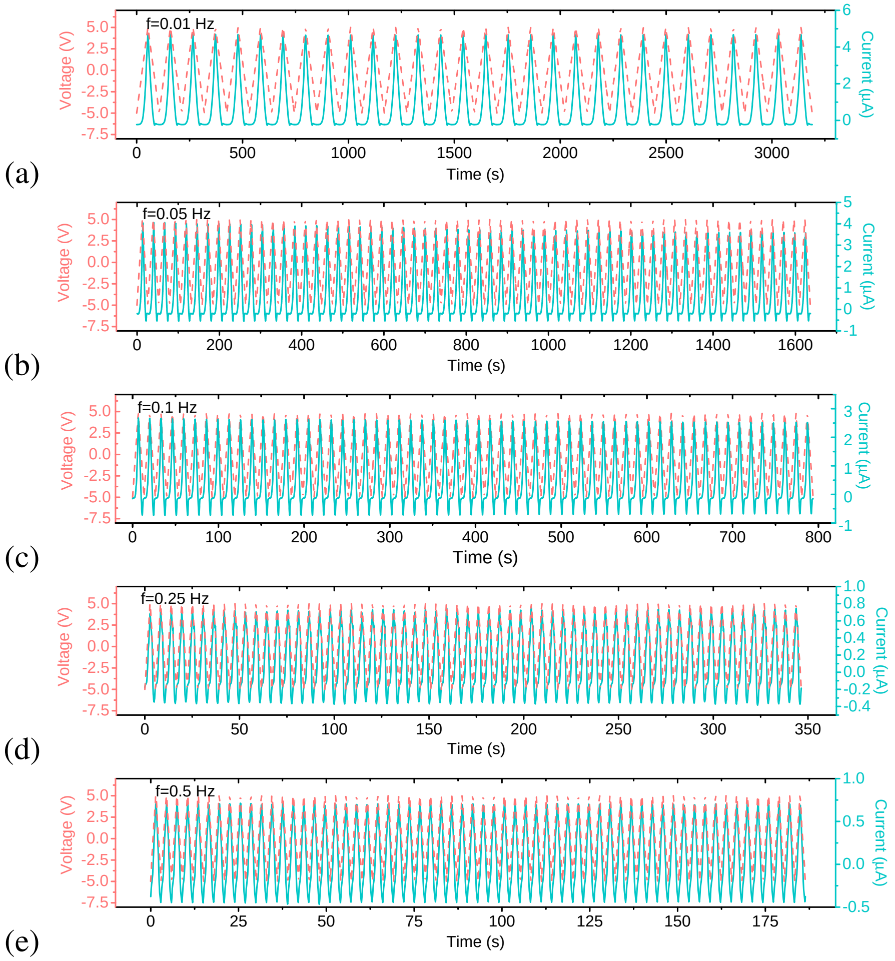

Figure S7: (a), (b), (c), (d) and (e) are the current recording in the experiments with scanning frequencies of 0.01Hz, 0.05Hz, 0.1Hz, 0.25Hz and 0.5Hz with amplitude of 5V. The devices can excellent repeat the I-V curves for over 30 cycles and up to 60 cycles at frequencies of 0.05Hz, 0.1Hz, 0.25Hz and 0.5Hz. shown in the manuscript, although we can found various type of current hysteresis in different specimens possibly due to the thin film properties at tip side.

2.5 Electrochemical measurement in different amplitude of voltage

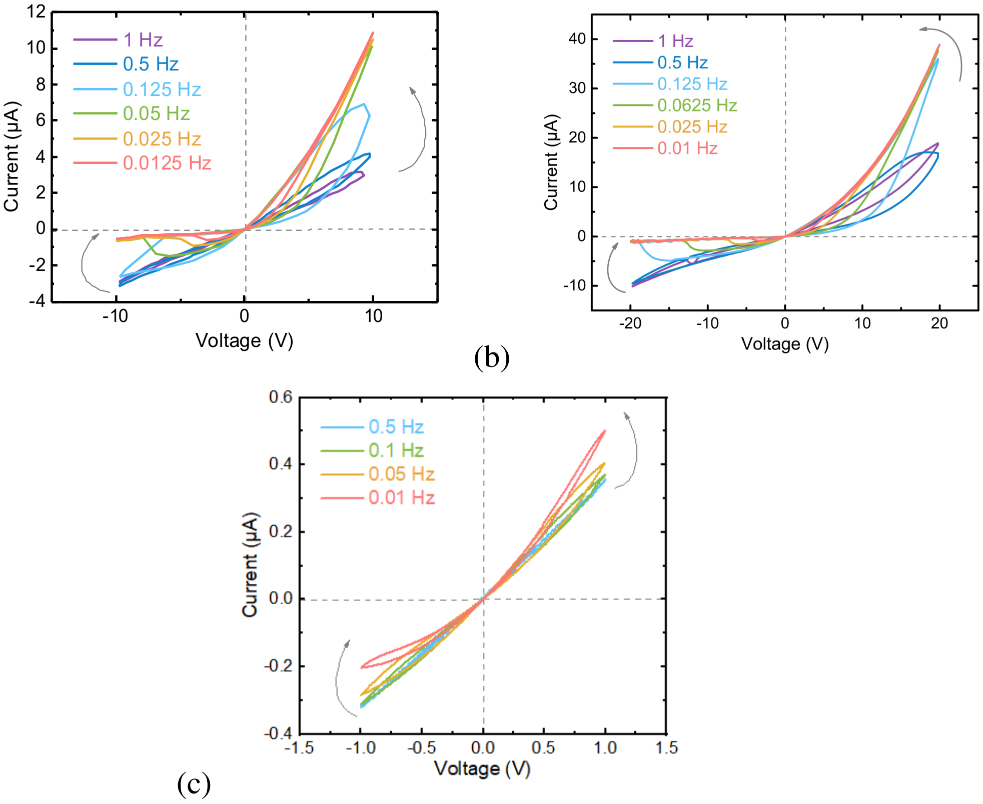

Figure S8: (a) the I-V curves with amplitude of scanning voltage of 10V, in various scanning frequencies. (b) the I-V curves with amplitude of scanning voltage of 20V, in various scanning frequencies. (c) The typical I-V curves with voltage amplitude of 1V in various scanning frequencies. Our results in amplitude of 1V didn't represents clear current hysteresis, illustrating that the amplitude of voltage or electrical field is critical for inducing the resistance switches in our nanofluidic memristor. The results shows that the limit of current in positive and negative bias voltage, possibly indicates the saturated strain of the tip. The represented results here is measured from the same specimens in the main text.

2.6 The impact of pH

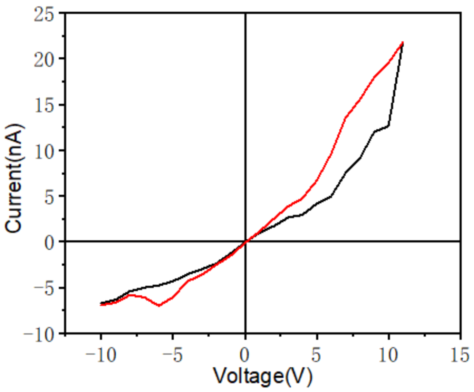

(a)

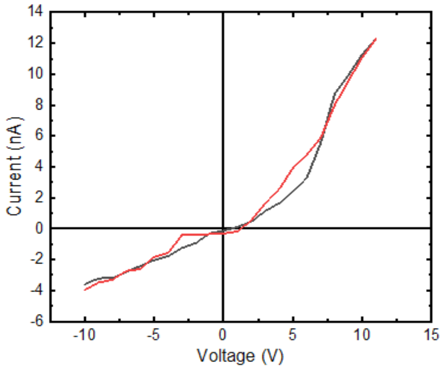

(b)

Figure S9: As we suspect the strain of nanopore tip side was activated by electrical force on the NPs. Hereby we measured the current responses under scanning frequencies of 0.025Hz in different solutions. (a) The current represents a current hysteresis at pH 8.5 solution, however not in pH3.5 solution(b) in the same frequencies. The measurements were measured in specimen 2.

2.7 The impact of particle size

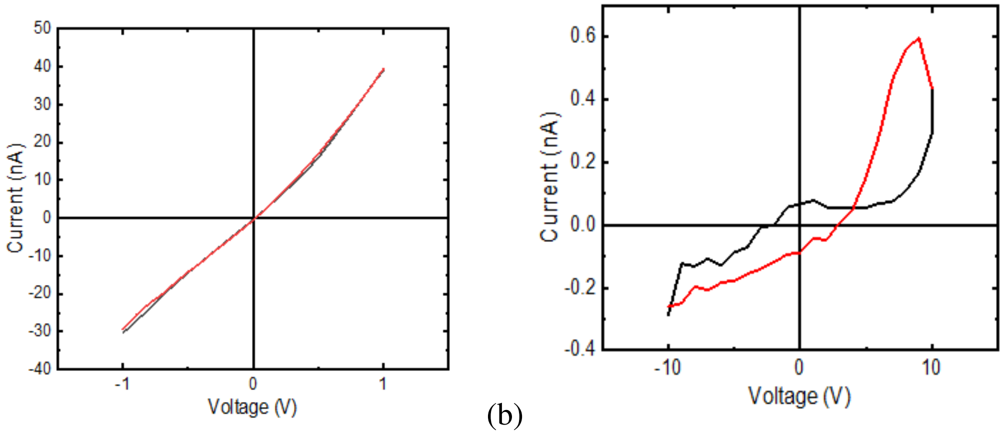

(a) (b)

Figure S10: To find the impact of particle size, we use the NPs with diameter of 100nm instead of the 15nm NPs in specimen 3. The figure (a) shows the typical I-V curves before adding NPs while figure (b) shows the typical I-V curves after NPs addictive. The resistance significantly increases with NPs, however still behave with a current hysteresis, again approving the key role of NPs in current hysteresis.

3 Numerical Simulations

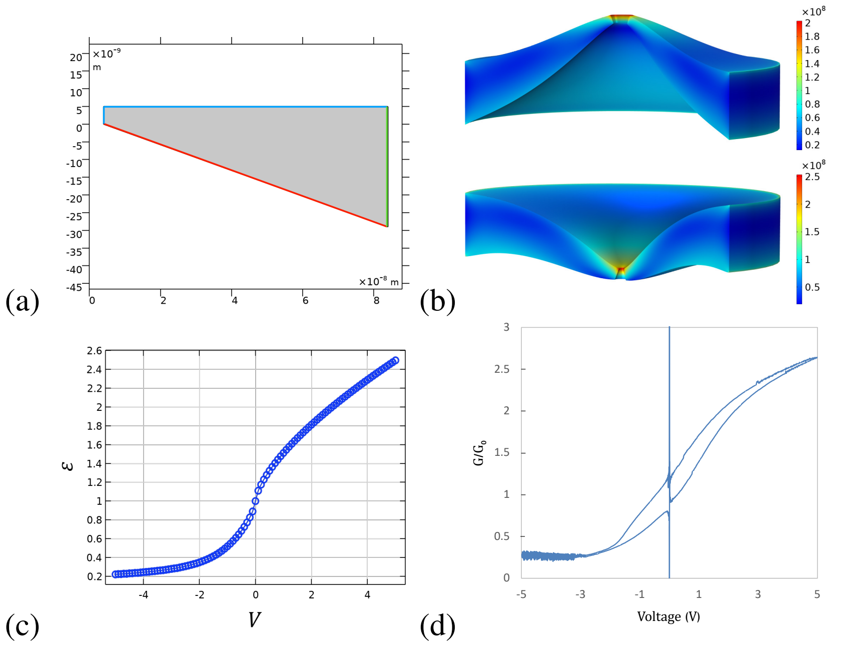

Figure S11: (a) schematically illustrated the tip of polymer channel in 1D axial system, with linear elastic model, when the stress is below a critical threshold value[4]. The blue lines indicate the free boundary of deformation. The load were applied at the inner surface of conical nanochannel marked in red. The boundary marked in green was a fixed. The correlation between stress and applied pressure. (b) shows the strain of tip under positive and negative pressure. The color indicates the stress distribution on the tip side of nanochannel. As can be seen, the deformation is limited at the negative applied pressure, since the roughness at the circumstance of tip may resist a perfect close of the channel. Thus, as we found in the experiments, we have a smallest value of the conductance at negative voltage (~ 0.1). To mimic this process, we set a stress of $\sigma/\sigma_0 \sim e^{-d/d_0}$, where $d_0 = 7.93nm$ is a critical diameter that resists further deformation. Finally, we could account the position and calculate the strain of polymer tip as a function of applied voltage shown in (c) as the solution in equilibrium states (or saying in $\Delta t/\tau \gg 1$), which matched well with the experimental results with frequency of 0.005Hz and amplitude of 5V.

References and Notes

[1] Apel P, Korchev Y, Siwy Z *et al.* Diode-like single-ion track membrane prepared by electro-stopping. *Nuclear Instruments and Methods in Physics Research Section B: Beam Interactions with Materials and Atoms* 2001; **184**: 337–346.

[2] Siwy Z, Apel P, Dobrev D *et al.* Ion transport through asymmetric nanopores prepared by ion track etching. *Nuclear Instruments and Methods in Physics Research Section B: Beam Interactions with Materials and Atoms* 2003; **208**: 143–148.

[3] Stein D, Kruithof M and Dekker C. Surface-Charge-Governed Ion Transport in Nanofluidic Channels. *Physical Review Letters* 2004; **93**: 035901.

[4] Denardin ELG, Tokumoto S and Samios D. Stress–strain behaviour of poly(ethylene terephthalate) (PET) during large plastic deformation by plane strain compression: the relation between stress–strain curve and thermal history, temperature and strain rate. *Rheologica Acta* 2005; **45**: 142–150.
